# Assessing *Mbiotisho*: A smartphone application used to collect high‐frequency health and nutrition data from difficult‐to‐reach populations

**DOI:** 10.1111/mcn.13496

**Published:** 2023-03-06

**Authors:** Nathaniel Jensen, Watson Lepariyo, Vincent Alulu, Simbarashe Sibanda, Beatrice N. Kiage

**Affiliations:** ^1^ Sustainable Livestock Systems International Livestock Research Institute Nairobi Kenya; ^2^ Nutrition Sensitive Agriculture Food, Agriculture and Natural Resources Policy Analysis Pretoria South Africa; ^3^ Department of Human Nutrition and Dietetics Jomo Kenyatta University of Agriculture and Technology Juja Kenya

**Keywords:** anthropometry, cost and cost analysis, data quality, digital data collection, epidemiological methods, health and nutrition data, self‐reported data, surveys and questionnaires

## Abstract

There is an urgent need for improved and timely health and nutrition data. We developed and tested a smartphone application that caregivers from a pastoral population used to measure, record and submit high‐frequency and longitudinal health and nutrition information on themselves and their children. The data were assessed by comparing caregiver‐submitted measurements of mid–upper arm circumference (MUAC) to several benchmark data sets, including data collected by community health volunteers from the participating caregivers during the project period and data generated by interpreting photographs of MUAC measurements submitted by all participants. We found that the caregivers participated frequently and consistently over the 12‐month period of the project; most of them made several measurements and submissions in at least 48 of the 52 weeks of the project. The evaluation of data quality was sensitive to which data set was used as the benchmark, but the results indicate that the errors in the caregivers' submissions were similar to that of enumerators in other studies. We then compare the costs of this alternative approach to data collection through more conventional methods, concluding that conventional methods can be more cost‐effective for large socioeconomic surveys that value the breadth of the survey over the frequency of data, while the alternative we tested is favoured for those with objectives that are better met by high‐frequency observations of a smaller number of well‐defined outcomes.

## Key messages

- Caregivers, even those that are illiterate and with little smartphone experience, can and will use a simple icon‐based smartphone application to measure, record and submit health and nutrition information on themselves and their children consistently and at high frequency.
- The errors in the data submitted by the caregivers are similar to those of data submitted by enumerators in similar settings.
- The cost per data point of submissions by caregivers is much lower than data collected using conventional approaches but the simple icon‐ and audio‐based approach to data collection may not be appropriate for all data needs.

## Graphical abstract

Health and nutrition data are extremely expensive to collect among remote or otherwise difficult‐to‐reach populations. We find that caregivers in a remote setting can and will collect and submit high‐frequency health and nutrition information on themselves and their children using the *Mbiotisho* smartphone application. This low‐cost approach to data collection offers many exciting opportunities for those working on health and nutrition issues in remote settings.

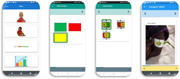

## 1 INTRODUCTION

Malnutrition is one of the main causes of child mortality and morbidity in many low‐ and middle‐income countries. Malnutrition in all its forms has harmful effects on human health and social development throughout the life cycle (Batis et al., 2020). In early childhood, undernutrition can have life‐long, irreversible consequences for a child's physical health and cognitive development (Black et al., 2013). Recent global trends indicate that 20.5 million new‐borns have a low weight at birth, one in five 5‐year olds is stunted and 45.5 million are wasted (Development Initiatives and Bristol, 2021). In most countries, progress to achieve global nutrition targets by 2025 is insufficient (Development Initiatives and Bristol, 2021) and, as has been stated repeatedly by the Global Nutrition Report, improving data is one critical step in reducing malnutrition (Development Initiatives, 2017, 2018, 2020, 2021).

Routine growth monitoring by clinicians and nutritionists is a common practice that helps to detect children at risk of malnutrition (Ashworth et al., 2008; Black et al., 2013; de Onis et al., 2004); direct essential resources when children's growth falters (Mangasaryan et al., 2011); track nutrition trends; and make child malnutrition more visible (Pearson and UNICEF, 1995). While growth monitoring may offer a promising foundation that could be built on to meet many data needs, routine monitoring relies on either bringing individuals to facilities or sending experts to communities. Unfortunately, these practices are expensive and often increase in cost in more remote locations, which is also where many malnourished people live. Further, as the recent COVID‐19 pandemic highlighted in many regions, this approach to monitoring is vulnerable on several fronts; for example, to disruptions in transportation networks, to migration, to conflict and to disease.

Community‐based monitoring, which relies on community members to perform assessments in the community, has proven to be an effective and low‐cost alternative to conventional monitoring approaches for diagnosing and referring individual cases of malnutrition. However, the measurements during community‐based monitoring are rarely recorded or structured to be used beyond the immediate diagnosis. Indeed, click‐mid–upper arm circumference (MUAC) (Michaels and Pearce, 2017), which inspired some of the approaches used by this research, is an example of successful innovation supporting the community‐based diagnosis of malnutrition, but that does not generate structured data. When data are recorded, their value is often limited by poor coverage, obscure and inconsistent sampling processes, and long delays between measurement and dissemination (Ashworth et al., 2008; Barnett and Gallegos 2013; Morley, 1994). Consequently, the full potential of such data, which could be used in many of the ways that conventional growth monitoring data are used (e.g., tracking malnutrition rates and impact evaluations), is often unmet. The result is that, when stakeholders need data, for example, to assess levels of malnutrition or to test the efficacy of an intervention, they often set up short‐term data collection structures that run parallel to existing community‐based monitoring infrastructure, which is both inefficient and does little to support the larger data ecosystem or local data collection capacity.

Our objective was to reduce the health and nutrition data gap by developing cost‐effective and scalable technologies to improve the collection and availability of high‐resolution (individual‐level) nutrition and health data, especially among populations that are typically underrepresented in existing monitoring and response mechanisms. To meet this objective, we invested in approaches that allow individuals to collect and submit information on themselves, their household members and the community. Adjusting from the standard model that relies on importing external data collectors to make measurements to a model that uses in situ data collectors, comes with large savings related to scheduling, staff time, enumerator wages and transportation. Reducing these costs, which are often substantial, creates opportunities to collect high‐frequency, short recall‐period data in situations where such data have historically been cost‐prohibitive to collect. This is not to say that such a model can always provide the same types of data that are generated by the conventional enumerator/respondent model. Rather, this research agenda aims to understand if our proposed alternative approach to the collection of data could be useful for specific types of data.

To test if this alternative model for data collection is useful, we developed a smartphone‐based tool that participants can use to measure, record, and submit information on their health and nutrition, and that of their children. The tool was designed explicitly to meet the data needs where they are greatest—that is, in difficult‐to‐reach populations with a high risk of malnutrition—although its application can be broad if properly validated. To be relevant, the tool must be usable by individuals of all backgrounds, and, therefore, was developed to be used without requiring previous experience with smartphones, literacy, numeracy, or connectivity. We then launched the tool among caregivers in several pastoral communities in Samburu County, Kenya, where low population densities and poor infrastructure have meant little access to services, and sustained high rates of global acute malnutrition (16%) have contributed to stunting in over 30% of the children under 5 years (Kenya National Bureau of Statistics KNBS, 2015).

The data submitted by participants were used to answer three research questions:

1. Can and will individuals consistently measure, record and submit information on diet, nutrition and health?
2. How do the measurements that individuals self‐report compare with those collected through more conventional approaches to field data collection?
3. Are the self‐report measurements collected at a lower cost than conventional approaches?

The tool that we developed and tested is one of many digital tools being proposed within the nutrition community to fill a host of gaps related to data collection and information dissemination. For example, the UNSCN's 45th volume of their publication *Nutrition*, was completely dedicated to the ways that digital technologies are impacting nutrition. The volume included several manuscripts that described digital technologies that aimed to address a wide range of data and information gaps, from training frontline workers through Elearning platforms (Sarkar et al., 2020), to providing customised health and nutrition information to individuals through mNutrition platforms (Barnett et al., 2020). Indeed, the digital tool that is being assessed in the research described by this manuscript was first described in that volume (Jensen et al., 2020). What sets our proposed solution apart from other proposals, is that ours is the only solution that was designed explicitly to address data gaps among those that are often underrepresented by conventional approaches because they are remote or otherwise difficult to monitor.

## 2 METHODS

### 2.1 Study design and implementation

**Figure 1:**
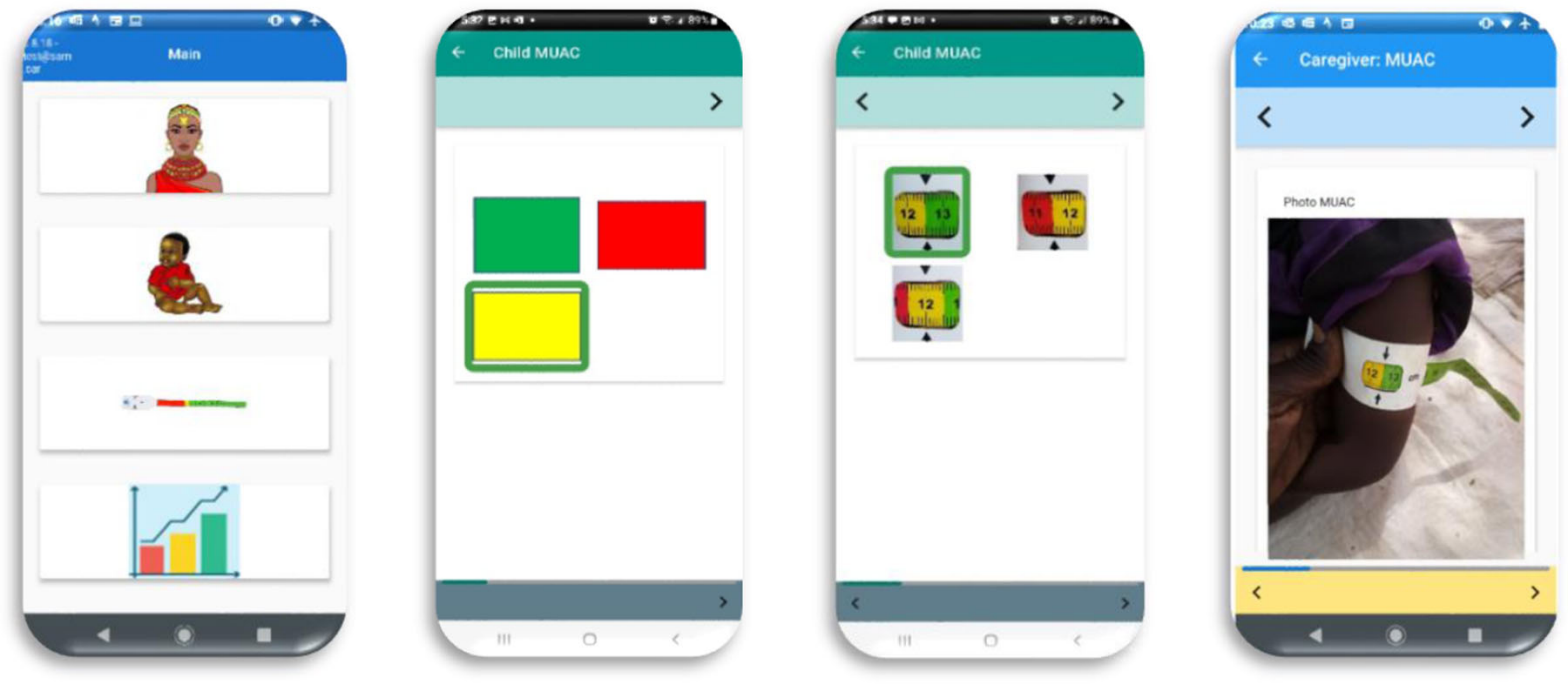
Screen shots from the *Mbiotisho* application. (a) (left side) is the landing screen. (b) (middle left) illustrates the first step of the MUAC process, in which the caregiver selects the colour of the measurement. (c) (middle right) illustrates how the caregiver then selects the measurement from the list of options that are consistent with the colour selected. (d) (right side) shows the photo of the measurement taken by the caregiver and submitted as part of the MUAC process. MUAC, mid–upper arm circumference.

To meet the research objectives, we worked with stakeholders and participants to develop the *Mbiotisho* smartphone application, which was to be used by caregivers to record information on their health and nutrition and that of an index child. *Mbiotisho* is a Samburu word that roughly translates to English as ʻour healthʼ. The application was designed to have a simple and intuitive user interface that does not require literacy or numeracy; the application uses icons and audio to communicate questions and record responses. Figure 1a (left side) includes an example of the application's landing screen, where the user selects one of the following processes.

**Caregiver update**: This process contains a sequence of questions on morbidity, food groups consumed, coping strategies employed and health‐seeking behaviour, all using a 24‐h reference period and all in reference to the participant.

**Child update**: This process is similar to the Caregiver Update but all questions are in reference to the index child.

**Child MUAC**: This process includes a sequence of screens facilitating the measurement and recording of the index child's MUAC and is illustrated in the right three panels of Figure 1.

**Tracking and feedback**: This feature moves through a sequence of screens providing the caregiver with a report on her recent submissions and customised references to international recommendations.

The Caregiver Update and the Child Update processes could be completed as frequently as once every 23 h, and the MUAC process as frequently as once every 6 days. If the participant attempts to complete any of the tasks more frequently, the application displays a screen and audio message thanking them for already having completed the process for that period. The Tracking and Feedback feature could be accessed as frequently as the caregiver liked.

**Figure 2:**
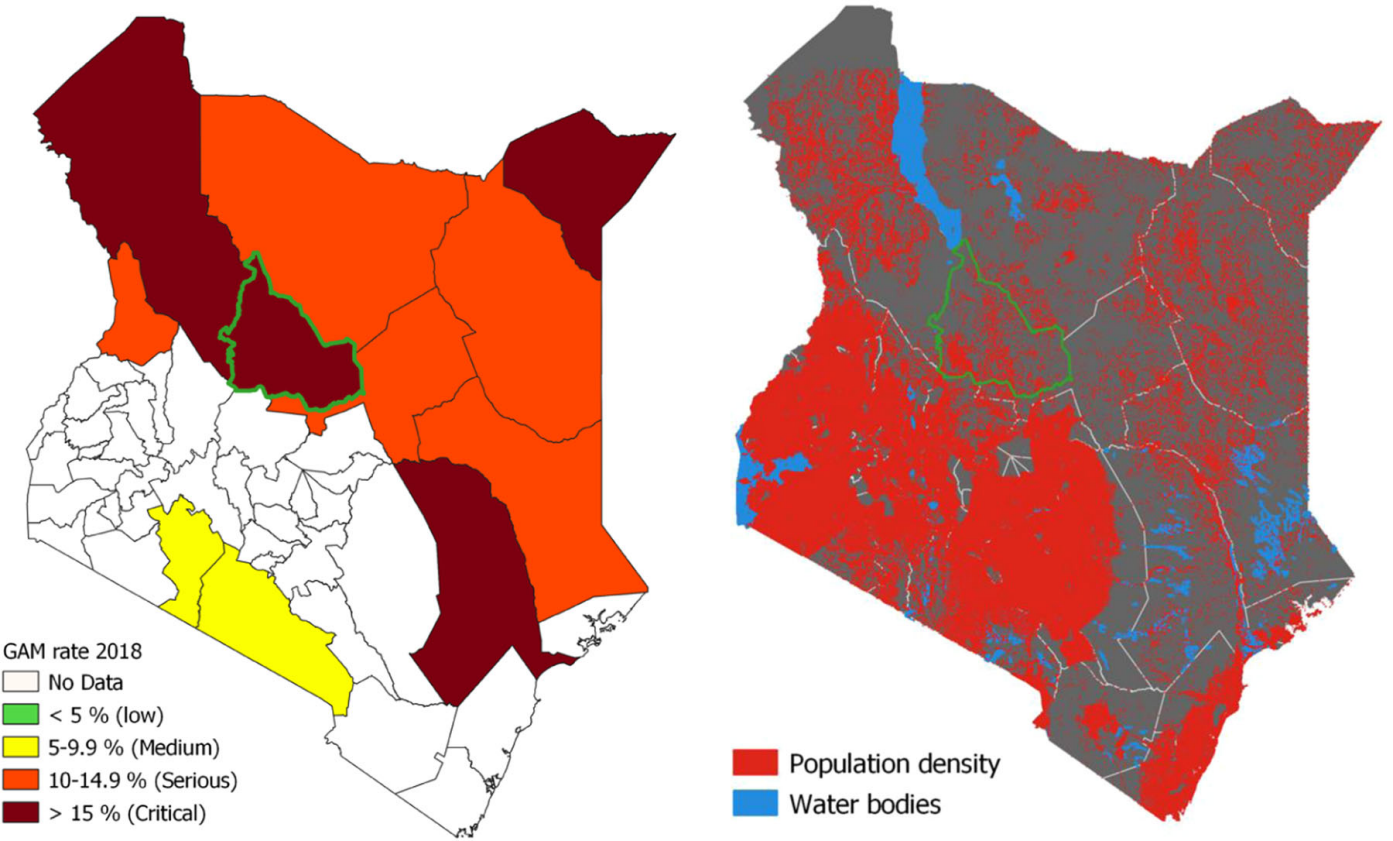
The study site—Samburu County—is highlighted in green in maps of Kenya, showing the rate of global acute malnutrition (GAM) in 2018 in the left panel (Kenya Ministry of Health, 2018) and population density in the right panel (Macharia et al., 2021).

Once the *Mbiotisho* application was developed, we then identified the pastoral regions of Samburu County in Kenya as the sampling frame because it contained a population with high rates of child undernutrition (Figure 2, left panel) and that present a challenge for typical and alternative approaches to surveillance; individuals that lived far from population centres (Figure 2, right panel), who sometimes migrated, and had low rates of literacy or familiarity with smartphones.

In November 2019, 22 Community Health Volunteers (CHVs) from four Community Health Units (CHUs) were selected by Samburu's Community Health Extension Workers (CHEW). These 22 CHVs were trained on the project's objective, and its indicators, on a survey tool developed by the project for the caregivers to use (hereinafter CHV Tool) and the participants' tool—*Mbiotisho*.

The CHVs were then asked to recruit nine participants from their respective catchment populations. The eligibility criteria were that the participants should be of childbearing age and the primary guardian of at least one child between the ages of 5 and 47 months at the time of recruitment. This age range was selected so that the children would be between 6 and 60 months during the 12 months of data collection. For the remainder of the study, the CHVs were asked to use the CHV tool to collect data from the participants (hereinafter caregivers) during their monthly visits. In theory, the CHV survey was only a minor additional burden to the CHVs, who were already meeting with caregivers each month as part of their duties to provide education on neonatal/infant care and to perform child health and nutrition monitoring. The CHVs were paid KES 3000 (approximately USD 30) during the project period per month by the project, which was to compensate them for the additional time required to collect the CHV survey from each of their caregivers each month and to incentivize their consistent participation.

Once recruited, the caregivers were trained on the health and nutrition guidelines related to the indicators that they would be tracking, and then trained on how to measure and record them using the *Mbiotisho* application. The caregivers were provided with smartphones and solar chargers and were asked to participate in the project by submitting records as much or as little as they liked, within constraints provided by the application. The participants received an incentive of KES 20 (USD 0.2) for each submitted update and KES 30 (USD 0.3) for each submitted MUAC process. The maximum incentive that a participant could collect in one 7‐day period was KES 340 (USD 3.4). They also received data bundles and phone credit worth KES 500 (USD 5.0) each month.

The study lasted 14 months between November 2019 and December 2020.

### 2.2 Empirical strategies

Each of the three research questions required its own empirical strategy.

Research question 1: Can and will individuals consistently measure, record and submit information on their diet, nutrition and health?

To answer this question, we calculated rates and consistency of participation across the project period. The 12‐month duration of the project offered ample time for any initial excitement with the application to wear off and for long periods without direct contact between the participants and project staff. The rates and consistency of participation by the caregivers were compared to those of the CHVs, which provided a relevant benchmark for participation (i.e., by local and trained technicians).

To learn about participants' experiences using the application, we collected a survey from them at the end of the project. The endline survey was collected using a conventional in‐person, enumerator approach, and faced several challenges related to migration and restrictions put in place in response to the COVID‐19 pandemic, which resulted in reduced participation (even while the participants continued to submit data via the *Mbiotisho* application). Of the 189 project participants, 128 participated in the endline survey. The descriptive statistics from these endline data are used to illustrate several points related to participation.

Research question 2: How do the measurements that individuals self‐report compare with those collected through more conventional approaches to field data collection?

Data quality was assessed by calculating the technical error of measurement (TEM) of paired sets of measurements over time. The calculation is described by Equation (1), where ${\mathrm{TEM}}_{i,j}$ is the TEM between measurements in data sets i and j, $y_{i,t}$ is the outcome measurement from data set i in period t, $y_{j,t}$ is the outcome measurement found in data set j in period t and T is the number of periods with paired measurements in both i and j.

(1) $$TEM_{i,j}\;=\sqrt{\frac{\sum_{t=1}^{T}{\left(y_{i,t}-y_{j,t}\right)}^{2}}{2T}}.$$

These TEM calculations were conducted for each participant to assess the consistency of their data with that of their CHVs. The temporal units of the caregiver's data are harmonised with that of the CHVs using monthly means. For example, for each of the caregiver‐CHV combinations, we calculated the TEM of their paired monthly data, measurements in the case of CHVs and monthly means in the case of caregivers, on the same child over time. The mean TEM for each CHV across their caregivers was also calculated as an indicator of the quality of each CHV's data.

While both participants and CHVs collected measurements related to several indicators, we focused on child MUAC for the analysis of data quality. We did this because MUAC is proven and internationally recognised as a useful indicator of nutritional status (Aguayo et al., 2015; Bliss et al., 2018; Myatt et al., 2006) and health (Binns et al., 2015; Sachdeva et al., 2016), it is used in many contexts and by many organisations (e.g., UNICEF, WFP), and it is low‐cost to implement, meaning that there is a high potential for scalability. Further, and this characteristic of MUAC is quite important for the comparability of the caregiver and CHV data, the change in MUAC over time is limited by biological processes, which allows us to compare data that were not necessarily collected on exactly the same day. Other indicators, such as consumption over the previous 24 h or morbidity, could change drastically from 1 day to the next.

The TEM calculated from MUAC measurements submitted by each caregiver and CHV on each index child, informs on the variation in measurements between the two. However, prior research and our own field reports from this project highlight that it is unlikely that the measurements submitted by the CHVs are free from errors and, therefore, that the TEM rates are solely attributable to the caregivers' errors. We tested the CHV data for evidence of errors by calculating the implied average rate of change in MUAC between longitudinal observations—MUAC velocity—and then flagging unrealistic values. We used the threshold of ±0.7 mm/day to identify unrealistic changes in MUAC over time as evidence of errors in the measurements submitted by the CHVs. More details on the MUAC velocity approach and how the threshold was set are found in Supporting Information: Appendix A.

### 2.3 Photo‐interpretation measurements

If the CHV data are imperfect, these data are not an accurate benchmark by which to assess the caregivers' data. Two alternative data sets were created using photo interpretation of images (PIM), which were captured as part of the MUAC process and submitted with each measurement. This method is of particular interest because (1) it tested for the internal validity of submissions and, therefore, does not require benchmark data, which are not available in most cases, and (2) it can be used to identify and clean identified errors from the data, therefore, improving the resulting data set.

Photographs of the MUAC measurements were taken and submitted by all participants during each of the MUAC measuring processes. Images were screened and those with apparent mismeasurement (e.g., arm bent or MUAC tape in the wrong location) or poor image quality (e.g., out of focus, too dark to see) were dropped. PIM of MUAC from the subset of photographs with clear images of (seemingly) correctly measured MUAC were extracted by the authors of this manuscript. A total of 593 measurements were extracted from images submitted by CHVs and 1058 measurements were extracted from images submitted by caregivers.

The result of the PIM process is an alternative data set for the caregivers and the CHVs. We then used the PIM data as alternative benchmarks with which to assess data quality by calculating TEMs.

### 2.4 Cost analysis

Research question 3: Are the self‐report measurements collected at a lower cost than conventional approaches?

The costs and profiles of data collected through the *Mbiotisho* application were compared with two common data collection scenarios. The first scenario mimicked the work‐horse of impact evaluations; an annual survey collected by enumerators and performed twice over the study period (e.g., baseline and endline). The second scenario is more aligned with what many projects and surveillance networks would like to have but cannot afford; and repeated monthly data collected by enumerators for 12 months.

For all three scenarios—*Mbiotisho*, annual and monthly—we assumed that data were collected from the 189 households that participated in the actual study. We included all the direct field expenses related to hardware, software, transportation, lodging and food. We also included staff time for a direct supervisor of the fieldwork, assuming that the field supervisor was active for all training, retraining and at any time that the enumerators were active. We did not include expenses related to initial sampling, developing or piloting any of the tools, or staff time related to project management or research because these were assumed to be similar across the approaches. The line items and rates used in the cost comparison (e.g., enumerator wages = USD 30/day, vehicle costs = USD 1.07/km) drew on real expenses from data collected by our team between 2018 and 2022 in the same regions and among similar populations. The details of the budget estimates are described in greater detail in Supporting Information: Appendix B.

### 2.5 Ethical Considerations

This study was conducted according to the guidelines laid down in the Declaration of Helsinki and all procedures involving research study participants were approved by the Institutional Research Ethics Committee (Ref no: ILRI‐IREC2019‐15) of the International Livestock Research Institute (ILRI) and the National Commission for Science, Technology and Innovation of Kenya (Ref: No. NACOSTI/P/19/72940/31932). Verbal informed consent was obtained from all subjects due to the high prevalence of illiteracy in the study population. The approved informed consent form was read to participants in their local language. Participants then had the opportunity to read over the informed consent form if they wished. Participants that wished to participate, consented to the enumerator orally, and then signed or placed an ‘X’ on the (digital) consent form. After consenting to their own participation, participants had the opportunity to enrol a child that they were the guardian of, into the study. Guardians provided consent on behalf of minors orally, which was documented in the digital consent form.

## 3 RESULTS

In total, the CHVs recruited 189 caregivers for the study, which reflects that several CHVs did not have the target number of nine eligible caregivers in their catchment region. At registration, the caregivers' ages ranged from 15 to 61, with an average age of 27. The caregivers reported housewife (50%), livestock keeping (33%) and causal labour (12%) as their primary occupations. Only one participant reported a salaried position. All were responsible for at least one child, and the median participant was responsible for three children. Just over half of the caregivers had received no formal education and only 11% reported that they knew how to use a smartphone when the project started.

In the endline survey, participants reported that tasks took an average of less than 5 minutes to complete. Caregivers reported liking the MUAC (39%) and consumption food groups (36%) modules the most, and only four participants reported not liking modules; child MUAC (*n* = 3), because it could be a challenge, and the consumption modules (*n* = 1), because no food was provided. Participants reported spending the incentives on food (98%), clothes (59%), other household items (16%), livestock (10%), medical care (5%) and as remittances to relatives (3%). All participants that responded to the endline survey said that they would be willing to participate in the programme if it started again.

Research question 1: Can and will individuals consistently measure, record and submit information on their diet, nutrition and health?

The tool was rolled out across the four CHUs in October and November of 2019. By the end of November, the 22 CHVs and 189 caregivers participating in the project had been trained on their tools and were collecting data. The data collection continued through mid‐December 2020, at which time the project closed one CHU at a time.

**Figure 3:**
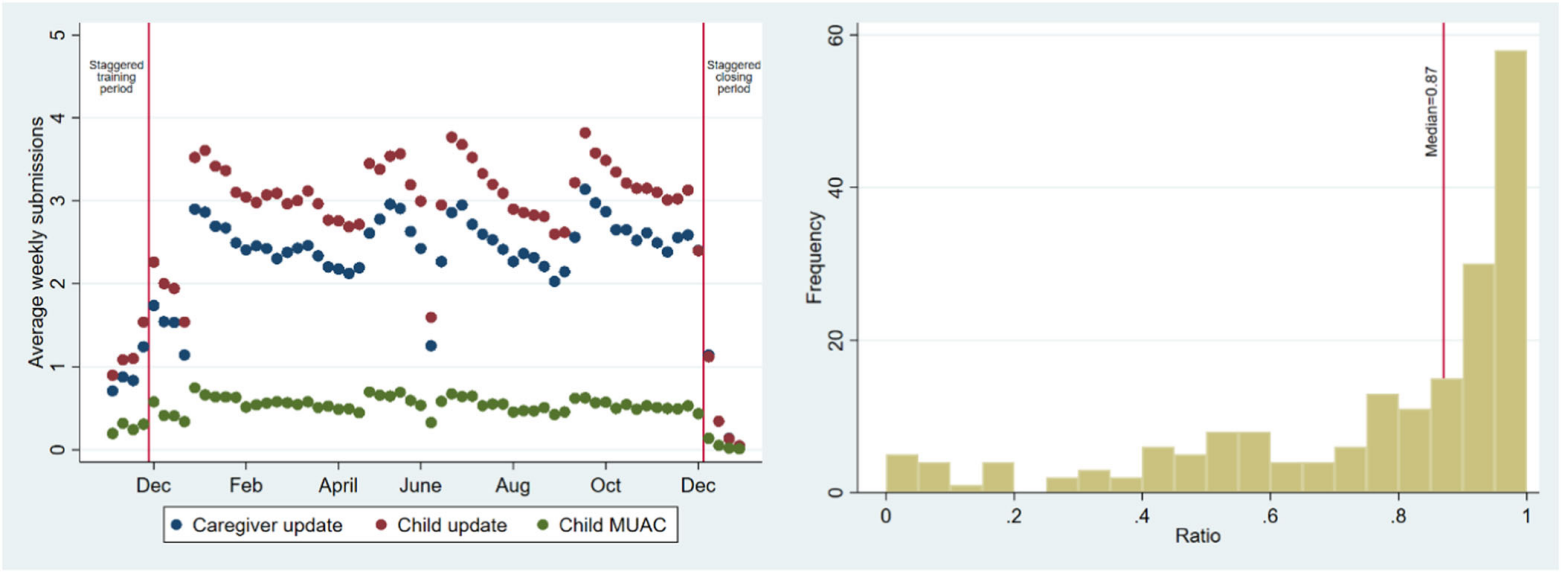
The left panel shows the average weekly submissions by caregivers during the implementation period. The right panel shows the ratio of the 52 active weeks in which each caregiver submitted data.

During the project period, the 189 participants collectively filed 63,687 submissions, comprising 25,788 caregiver updates, 32,090 child updates and 5809 child MUAC readings. The left panel of Figure 3 illustrates the weekly average submission rate across the 189 caregivers during the period of the project, by submission type.

There were periods with considerably lower participation, notably during the training and launch of the tool (November 2019), the period immediately after the launch during which participants frequently faced technical issues (December 2019), when an update to the application caused some technical issues with some devices (June 2020), and during the closing period (December 2020). The staggered launch and closing caused variation in participation that is mechanical, in that participants could not participate before the project launched or after it closed in their CHU. Therefore, we restricted the data from here forward to those collected during the 364 days (52 weeks) that all four CHUs were active; between and including December 1, 2019, and November 30, 2020. This period excludes the staggered activation and closing periods but includes those periods when there were challenges to the project.

The total average submission per week per contributor was 6.0. Note that the total maximum allowable submissions in a week from a participant were 16, which would be composed of seven daily submissions for each of the updates and two MUAC processes. For the data to be useful for individual‐level longitudinal analysis, frequent submissions must also be complemented by consistent participation. Using a weekly time step, we counted the number of weeks out of the total active data collection weeks that each household submitted any tasks. This ratio is illustrated for each of the 189 caregivers in the right panel of Figure 3, which shows that 50% of caregivers submitted data in at least 45 (87%) of the 52 active weeks and 30% of the caregivers submitted data in 95% of active weeks. Only 14 caregivers (7%), submitted less than 12 times, the equivalent of once a month during the active data collection. These figures include the three caregivers that did not make a single submission during the project's active data collection period.

We compared the participation rates of the caregivers with those of the CHVs, who were hired by the project to survey the caregivers each month. Forty‐five percent of the 22 CHVs participated at least once every month during the 12 months of the project and only one CHV participated in less than half of the months. At the same time, none of the CHVs submitted records for all the caregivers assigned to them each month and 50% of CHVs submitted less than 85% of the caregiver‐month observations that they were assigned, which is the equivalent of submitting a measurement for each caregiver in 10 of the assigned 12 months.

In summary, caregivers participated at rates and with consistency that is on par with, or better than, that of the CHVs, who were paid more to do an activity that should have been a very little additional burden on them because it was consistent with their existing obligations as CHVs. Indeed, the lower rate of participation by the CHVs undermined the value of their data as a benchmark, both because of the gaps it left in the benchmark and because it implied a certain lack of effort, which could also be reflected in the accuracy of their data.

Research question 2: How do the measurements that individuals self‐report compare with those collected through more conventional approaches to field data collection?

**Figure 4:**
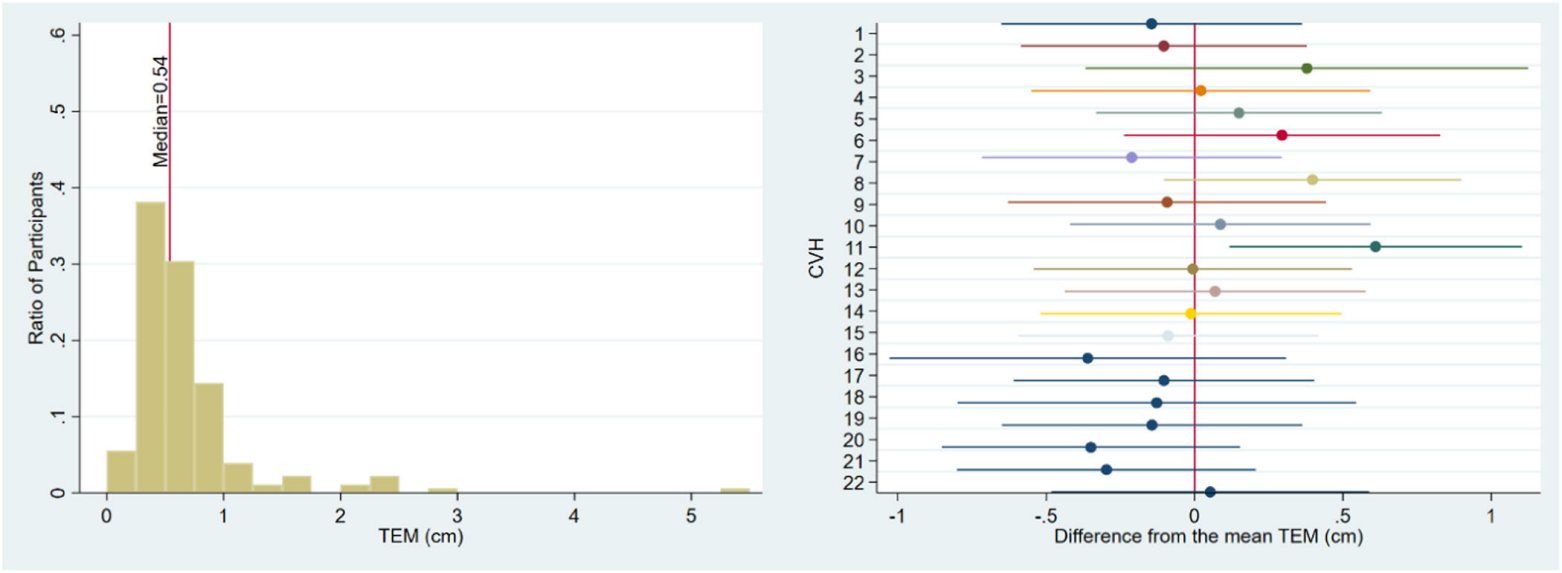
The left panel displays the distribution of TEMs for each of the 189 caregivers. The right panel displays the point estimates and 99% confidence intervals of the differences between each CHV's mean TEM across clients, and the overall mean TEM across all CHVs. CHV, Community Health Volunteer; TEM, technical error of measurement.

The TEMs were calculated for each longitudinal sequence of paired caregiver‐CHV measurements of child MUAC. Because each child was paired with an individual caregiver, the result is a TEM for each of the 181 caregivers for whom there was at least one caregiver‐CHV paired measurement. The left panel of Figure 4 includes a histogram of the TEMs for the 181 caregiver‐CHV pairs. There is a wide distribution of TEMs across the caregivers, ranging between 0.14 and 5.38 cm, with a mean of 0.68 cm. Most participants had a TEM below 0.55 cm.

To test for heterogeneity among CHVs, we compared the mean TEM for each CHV to the pooled mean of the TEMs related to the remaining 21 CHVs. The right panel of Figure 4 illustrates the point estimates and 99% confidence intervals of those differences for each CHV. CHV 011's mean TEM is 1.26 cm, which is nearly double the mean TEM of the remaining CHVs and is statistically greater at the 1% level (*t*‐stat = 3.22). CHV 011 had a TEM of greater than 2.0 cm for three of the nine caregivers that she worked with and greater than the pooled mean for six of the nine caregivers. While it is possible that many of the participants that CHV 011 worked with were submitting low‐quality data, such clustering is unlikely, given the observed distribution of TEMs across the other 21 CHVs and could equally be the result of errors submitted by CHV 011.

Testing for unrealistic MUAC velocities in the CHVs' data showed that 36% of CHVs violated the ±0.7 mm/day threshold at least once. See Supporting Information: Appendix A for the detailed analysis of MUAC velocity.

The fact that at least one CHV had average TEMs that were statistically significantly higher than their peers and that more than a third of the CHVs submitted at least one unrealistic sequence of measurements, suggests that the CHVs' data are imperfect. If so, the TEMs generated by the direct comparison of the caregiver and raw CHV data are not accurate methods for assessing the data submitted by the caregivers.

We, therefore, turn to the PIM data for an alternative benchmark. Before using the PIM data for assessing caregiver data quality, we first assessed its validity by comparing the CHV‐collected data with the PIM data generated from their own submissions (PIM‐CHV data henceforth). If the CHV data were reasonably accurate with a low rate of, but potentially meaningful, errors, as the TEM and velocity analyses suggested, then the TEM between the CHV data and the PIM‐CHV data should have been quite low but greater than zero. Indeed, the mean TEM across the 22 CHVs between these two data sets—CHV and PIM‐CHV—was 0.13, well within the values commonly found for intraobserver or interobserver measurements of MUAC (Ulijaszek and Kerr, 1999). In addition, there is not a single velocity violation in the PIM‐CHV data, which also points towards a measurable improvement in quality. A similar assessment of the caregiver data and PIM‐caregiver data showed a decline in the rate of velocity violations from 2.2% to 0.05% between the two data sets (Supporting Information: Appendix A).

Given that the PIM data have been shown to be of higher quality than the measurements recorded by the participants, at least with respect to MUAC velocity, the PIM data are a more appropriate benchmark than the measurements recorded directly by the CHVs. The median TEM falls from 0.54 cm (Analysis A, Table 1) to 0.44 cm (Analysis B, Table 1) when the caregiver data are compared with the PIM‐CHV data rather than the raw CHV data (Table 1). Because there is a loss in the number of caregivers represented in the PIM‐CHV data, the PIM‐caregiver data are also included as a second benchmark. The median TEM between the measurements recorded by caregivers and the PIM‐caregiver data is lower still, 0.23 cm (Analysis C, Table 1).

**Table 1:** Median TEM between the caregiver data and the comparison data after performing the described screening processes.

	Comparison data		
	CHV	PIM‐CHV	PIM‐Caregiver
*All caregiver measurements*			
Analysis	A	B	C^1^
Median TEM	0.54	0.44	0.23
Caregivers represented	181	146	148
Observations	1345	434	589
*Subsample of caregiver measurements accompanied by a photo of PIM quality*			
Analysis	D	E	F^1^
Median TEM	0.45	0.37	0.23
Caregivers represented	139	86	148
Observations	454	182	589

*Note*: Analysis C and F are identical by construction.

Abbreviations: CHV, Community Health Volunteer; PIM, photo‐interpretation of images; TEM, technical error of measurement.

The considerable improvement in TEMs when using the PIM data could originate in differences in measurements between each participant and the PIM data generated from their submissions or from a selection effect of the PIM process. Specifically, the PIM process excluded measurements that appeared to be performed incorrectly and those that were done in difficult conditions (e.g., dark, child‐moving) that resulted in poor image quality. We, therefore, also calculated the TEMs between the measurements made by caregivers that were accompanied by a photograph that was PIM quality—those in which the image was sufficiently clear and that appeared to be making a measurement correctly. The further improvements in TEMs between the subsample of caregiver data with PIM quality photographs and the CHV (Analysis D, Table 1) or PIM‐CHV data sets (Analysis E, Table 1) suggests that the PIM eligibility criterion alone is a powerful way for screening out poor‐quality submissions. However, there is clearly a drawback in that the sample size is reduced considerably in this subsample.

Research question 3: Are the self‐report measurements collected at a lower cost than conventional approaches?

We estimated the following total direct field costs of data collection for each of the three scenarios: $49,317 for the annual data collection by enumerators, $176,897 for the monthly data collection by enumerators and $125,066 for the high frequency (multiple submissions per participant per week) by caregivers through *Mbiotisho*. Because there is concern that providing devices to each household is cost prohibitive, making the *Mbiotisho* approach unviable, we highlight here that the costs of participants' phones and chargers were included in the estimates, and we assumed complete depreciation of those devices by the end of the implementation—we did not sell them to recoup sunk costs. The line items used to estimate the costs of each activity are provided in Supporting Information: Appendix C.

The cost per individual round of indicators under consideration were $128, $76 and $13 for the conventional annual (*N* = 189 caregivers × 2 rounds = 378), conventional monthly (*N* = 189 caregivers × 12 rounds = 2268) and *Mbiotisho* high‐frequency methods (*N* = 189 caregivers × 52 rounds = 9828), respectively. While there are likely to be cases in which the technical expertise and skills of the enumerator are required, we believe that there are also cases in which such expertise is not worth the cost of reduced data frequency or coverage. The lower costs per round of data through *Mbiotisho* expand the opportunities of the fixed budget. Determining which approach is best for a particular activity requires thinking through the objectives of data collection, the available budget, the cost of transportation, and the capacity of the local participants and prospective enumerators.

## 4 DISCUSSION

Our implementation in Samburu aimed at testing a low‐cost method for data collection among populations that are typically challenging to monitor. In this case, the women that participated in this study were extensive pastoralists, which also means that they live in regions with poor infrastructure and might migrate frequently, making them expensive to interview and difficult to locate for repeat visits.

Participation rates over the 12‐month project show clearly that caregivers are able and willing to use *Mbiotisho* to record and submit measurements, consistently, at high frequency, and over an extended period of time. Indeed, the participation rates and submission consistency among caregivers were on par with, or better than, that of the CHVs that were hired to collect benchmark data from the caregivers during their normal CHV activities. These findings both highlight issues related to conventional data collection that relies on enumerators or technicians to collect data, and the viability of *Mbiotisho* for data collection. Further, the participants continued to submit data without any change in quantity in the mid‐2020 period, during which policies meant to slow the spread of the COVID‐19 pandemic stopped nearly all in‐person field data collection in Kenya, illustrating that the tested approach can operate in some circumstances that are not appropriate for conventional ones.

Data quality is a serious concern for all field data collection. While several studies have found that caregivers can accurately use MUAC measurements to monitor malnutrition (e.g., Alé et al., 2016; Blackwell et al., 2015; Grant et al., 2018; Isanaka et al., 2019), it continues to be uncommon and, as with community‐based monitoring, there has been little work on developing methods for those caregivers to easily record their measurements in a structured way so that they can provide value beyond their immediate screening objective. Assessing the data submitted by caregivers and CHVs in this study, showed that both groups submitted data that contained errors. The errors in the measurements submitted by the CHVs highlight that all field data collected by humans, even that which has been collected by highly trained and experienced individuals, requires appropriate incentive structures and monitoring for quality. In this case, the CHVs' data quality was not well monitored and, in the end, could not be used as an accurate benchmark with which to assess the caregivers' data quality.

To develop an alternative benchmark, we employed a novel approach that used photographs of measurements submitted by participants to generate an alternative sequence of measurements. Assessments of data quality using the alternative benchmark were promising. The TEM between the caregiver data and the PIM‐CHV data was 0.44 cm, which is well within the range of 0.10–1.3 cm reported by Ulijaszek and Kerr (1999) in their review of 11 studies of anthropometric measurements, but above the reported averages across those studies (0.37 cm). When images of the measurements made by the caregivers were used to screen the caregivers' data and filter out what appear to be incorrect measurements, the median TEM fell to 0.37 cm. When the PIM‐caregivers' data were used as the benchmark, which could arguably be the most appropriate benchmark, the TEM fell to 0.23 cm. These figures are on par with those from trained enumerators. For example, in a six‐country assessment of the reliability of anthropometric measurements in the Multi‐Growth Reference Study, the WHO Multicentre Growth Reference Study Group (WHO Multicentre Growth Reference Study Group, 2006) found that the average TEM between their trained enumerators for MUAC ranged from 0.18 to 0.23 cm, even after employing procedures for reducing TEMs by triggering measurements when large differences between measures were observed.

Here, we note that it was common for individual measurements made by the caregivers to be noisy, but one advantage of high‐frequency longitudinal data is that errors can be temporally smoothed to mitigate the impacts of outliers, or those outliers can be identified and dropped. The success of these approaches in improving the quality of data is an important finding, highlighting the value of building methods for monitoring data quality in surveys when comparison data sets will not be available. Such strategies can be used to identify contributors that are submitting low‐quality data and then either retraining them or dropping them from the activities. Indeed, while the *Mbiotisho* tool does collect information on several other indicators, including consumption and morbidity, we did not assess them because those indicators offer fewer options for accurate testing—the survey contained few options for internal or intertemporal cross‐verification because the variables have short recall periods and can change quickly and frequently. At the same time, accurately measuring MUAC requires considerable skill, much more than what is required for accurately measuring the other indicators collected by our tools.

With the identified approaches to screening data, we can generate data sets of high quality from the caregiver‐measured and ‐submitted data. While the approach does require expenses related to initial training in the same way that standard household surveys require for enumerators, this alternative approach avoids many of the other costs related to standard household survey approaches, such as transporting, feeding and lodging enumerators and field supervisors. For those with objectives that can be best met by high‐frequency data on a few indicators, the methods tested here have proved to be cost‐effective. If one's objectives require a large number of indicators or indicators that require a great deal of technical expertise, and the frequency of the data is less important, then conventional surveys are likely to be more cost‐effective.

The *Mbiotisho* tool and the methods of data collection that it employs hold a clear value for situations in which conventional data collection is challenging and/or expensive, in which frequent repeat measurements are valued, and in which the focus is on a few outcomes that can be translated into simple survey questions. We see several such situations and are employing *Mbiotisho* as part of surveillance networks for remote regions, for monitoring individuals between clinic visits, and among those living in informal settings. Data redundancies are being built into these activities so that we can continue to learn about which types of data individuals can and will collect consistently and accurately. We are also experimenting with incentive structures to test if the cost of data can be reduced further and if the quality of data can be improved. Our hope is to use the current implementations to further develop and improve the *Mbiotisho* tool and related processes, and then offer it openly for others to use.

## AUTHOR CONTRIBUTIONS

Nathaniel Jensen, Watson Lepariyo, Vincent Alulu, and Simbarashe Sibanda contributed equally to the development of the study and related tools and research design. Watson Lepariyo and Vincent Alulu led the field implementation of the study with support from Nathaniel Jensen and Simbarashe Sibanda. Nathaniel Jensen led the analysis and manuscript development with support from Watson Lepariyo, Vincent Alulu, Simbarashe Sibanda, and Beatrice N. Kiage.

## CONFLICT OF INTEREST STATEMENT

The authors declare no conflict of interest.

## Supporting information

Supporting information.

## Data Availability

Data that support the findings of this study are available from the corresponding author upon reasonable request.

## References

[mcn13496-bib-0001] Aguayo, V. M. , Aneja, S. , Badgaiyan, N. , & Singh, K. (2015). Mid upper‐arm circumference is an effective tool to identify infants and young children with severe acute malnutrition in India. Public Health Nutrition, 18(17), 3244–3248. 10.1017/S1368980015000543 25757562 PMC10271291

[mcn13496-bib-0002] Alé, F. G. B. , Phelan, K. P. Q. , Issa, H. , Defourny, I. , Le Duc, G. , Harczi, G. , Issaley, K. , Sayadi, S. , Ousmane, N. , Yahaya, I. , Myatt, M. , Briend, A. , Allafort‐Duverger, T. , Shepherd, S. , & Blackwell, N. (2016). Mothers screening for malnutrition by mid‐upper arm circumference is non‐inferior to community health workers: Results from a large‐scale pragmatic trial in rural Niger. Archives of Public Health, 74(1), 38. 10.1186/s13690-016-0149-5 27602207 PMC5011948

[mcn13496-bib-0003] Ashworth, A. , Shrimpton, R. , & Jamil, K. (2008). Growth monitoring and promotion: Review of evidence of impact. Maternal & Child Nutrition, 4, 86–117. 10.1111/j.1740-8709.2007.00125.x PMC6860476 18289158

[mcn13496-bib-0004] Barnett, I. , & Gallegos, J. V (2013). *Using mobile phones for nutrition surveillance: A review of evidence. IDS evidence report*. (IDS Evidence Report; 1).

[mcn13496-bib-0005] Barnett, I. , Gordon, J. , Faith, B. , Hidrobo, M. , Palloni, G. , Batchelor, S. , Scott, N. , & Gilligan, D. O (2020). Using mobile‐phone technology to change behaviour: Lessons from MNutrition. UNSCN.

[mcn13496-bib-0006] Batis, C. , Mazariegos, M. , Martorell, R. , Gil, A. , & Rivera, J. A. (2020). Malnutrition in all its forms by wealth, education and ethnicity in Latin America: Who are more affected?” Public Health Nutrition, 23(S1), s1–s12. 10.1017/S136898001900466X 32900396 PMC10200386

[mcn13496-bib-0007] Binns, P. , Dale, N. , Hoq, M. , Banda, C. , & Myatt, M. (2015). Relationship between mid upper arm circumference and weight changes in children aged 6–59 months. Archives of Public Health, 73(1), 54. 10.1186/s13690-015-0103-y 26693279 PMC4685635

[mcn13496-bib-0008] Black, R. E. , Victora, C. G. , Walker, S. P. , Bhutta, Z. A. , Christian, P. , de Onis, M. , Ezzati, M. , Grantham‐McGregor, S. , Katz, J. , Martorell, R. , & Uauy, R. (2013). Maternal and child undernutrition and overweight in low‐income and middle‐income countries. The Lancet, 382(9890), 427–451. 10.1016/S0140-6736(13)60937-X 23746772

[mcn13496-bib-0009] Blackwell, N. , Myatt, M. , Allafort‐Duverger, T. , Balogoun, A. , Ibrahim, A. , & Briend, A. (2015). Mothers understand and can do it (MUAC): A comparison of mothers and community health workers determining mid‐upper arm circumference in 103 children aged from 6 months to 5 years. Archives of Public Health, 73(1), 26. 10.1186/s13690-015-0074-z 25992287 PMC4436117

[mcn13496-bib-0010] Bliss, J. , Lelijveld, N. , Briend, A. , Kerac, M. , Manary, M. , McGrath, M. , Weise Prinzo, Z. , Shepherd, S. , Marie Zagre, N. , Woodhead, S. , Guerrero, S. , & Mayberry, A. (2018). Use of mid‐upper arm circumference by novel community platforms to detect, diagnose, and treat severe acute malnutrition in children: A systematic review. Global Health: Science and Practice, 6(3), 552–564. 10.9745/GHSP-D-18-00105 30185435 PMC6172115

[mcn13496-bib-0011] de Onis, M. , Wijnhoven, T. M. A. , & Onyango, A. W. (2004). Worldwide practices in child growth monitoring. The Journal of Pediatrics, 144(4), 461–465. 10.1016/j.jpeds.2003.12.034 15069393

[mcn13496-bib-0012] Development Initiatives . (2017). Nourishing the SDGs.

[mcn13496-bib-0013] Development Initiatives . (2018). Shining a Light to Spur Action on Nutrition.

[mcn13496-bib-0014] Development Initiatives . (2020). Action on Equity to End Malnutrition.

[mcn13496-bib-0015] Development Initiatives . (2021). The State of Global Nutrition.

[mcn13496-bib-0016] Grant, A. , Njiru, J. , Okoth, E. , Awino, I. , Briend, A. , Murage, S. , Abdirahman, S. , & Myatt, M. (2018). Comparing performance of mothers using simplified mid‐upper arm circumference (MUAC) classification devices with an improved MUAC insertion tape in Isiolo County, Kenya. Archives of Public Health, 76(1), 11. 10.1186/s13690-018-0260-x 29484177 PMC5822476

[mcn13496-bib-0017] Isanaka, S. , Berthé, F. , Nackers, F. , Tang, K. , Hanson, K. E. , & Grais, R. F. (2020). Feasibility of engaging caregivers in at‐home surveillance of children with uncomplicated severe acute malnutrition. Maternal & Child Nutrition, 16(1), 12876. 10.1111/mcn.12876 PMC7038908 31336045

[mcn13496-bib-0018] Jensen, N. D. , Alulu, V. , Lepariyo, W. , Madzivhandila, T. , Mkandawire‐Munthali, B. , & Sibanda, S. (2020). Improving nutrition and health data to and from remote regions. United Nations System Standing Committee on Nutrition (UNSCN)−Nutrition, 45, 96–102.

[mcn13496-bib-0019] Kenya Ministry of Health . (2018). *Nutrition standardized monitoring and assessment of relief and transitions (SMART) survey data*, 2018.

[mcn13496-bib-0020] Kenya National Bureau of Statistics (KNBS) . 2015. *Kenya Demographic and Health Survey (DHS), 2014*. KNBS and The DHS Program/ICF International.

[mcn13496-bib-0021] Macharia, P. M. , Mumo, E. , & Okiro, E. A. (2021). Modelling geographical accessibility to urban centres in Kenya in 2019. PLoS One, 16(5), e0251624. 10.1371/journal.pone.0251624 33989356 PMC8127925

[mcn13496-bib-0022] Mangasaryan, N. , Arabi, M. , & Schultink, W. (2011). Revisiting the concept of growth monitoring and its possible role in community‐based nutrition programs. Food and Nutrition Bulletin, 32(1), 42–53. 10.1177/156482651103200105 21560463

[mcn13496-bib-0023] Michaels, R. E. , & Pearce, J. M. (2017). 3‐D printing open‐source Click‐MUAC bands for identification of malnutrition. Public Health Nutrition, 20(11), 2063–2066. 10.1017/S1368980017000726 28488563 PMC10261415

[mcn13496-bib-0024] Morley, D. (1994). Will growth monitoring continue to be part of primary health care?” South African Medical Journal July, Suppl, 15–16. 7839185

[mcn13496-bib-0025] Myatt, M. , Khara, T. , & Collins, S. (2006). A review of methods to detect cases of severely malnourished children in the community for their admission into Community‐Based therapeutic care programs. Food and Nutrition Bulletin, 27(3_suppl3), S7–S23. 10.1177/15648265060273S302 17076211

[mcn13496-bib-0026] Pearson, R. , & UNICEF . (1995). Thematic evaluation of UNICEF support to growth monitoring. UNICEF.

[mcn13496-bib-0027] Sachdeva, S. , Dewan, P. , Shah, D. , Malhotra, R. K. , & Gupta, P. (2016). Mid‐upper arm circumference v. Weight‐for‐Height Z‐Score for predicting mortality in hospitalized children under 5 years of age. Public Health Nutrition, 19(14), 2513–2520. 10.1017/S1368980016000719 27049813 PMC10271052

[mcn13496-bib-0028] Sarkar, A. , Bader, N. , Khara, N. , Jain, N. , Anjem Mir, U. , Sharma, K. , & Qualitz, G. (2020). ELearning to empower Front‐Line nutrition workers in India. United Nations System Standing Committee on Nutrition (UNSCN)−Nutrition, 45, 49–56.

[mcn13496-bib-0029] Ulijaszek, S. J. , & Kerr, D. A. (1999). Anthropometric measurement error and the assessment of nutritional status. British Journal of Nutrition, 82(3), 165–177. 10.1017/S0007114599001348 10655963

[mcn13496-bib-0030] WHO Multicentre Growth Reference Study Group . (2006). Reliability of anthropometric measurements in the WHO multicentre growth reference study. Acta Paediatrica, 95(S450), 38–46. 10.1111/j.1651-2227.2006.tb02374.x 16817677

