# Supplementary material for: Assessing Mbiotisho: A smartphone application used to collect high‐frequency health and nutrition data from difficult‐to‐reach populations
Source: Matern Child Nutr. 2023 Mar 6;19(3):e13496. doi: 10.1111/mcn.13496 (PMC10262886; doi:10.1111/mcn.13496)
Supplement: Supplementary file 1 — Supporting information. [file MCN-19-e13496-s001.docx]

Supplementary appendices for the manuscript titled “Assessing Mbiotisho: A smartphone application used to collect high-frequency health and nutrition data from difficult to reach populations”

# Appendix A: MUAC Velocity

MUAC velocity is a calculation of the change in MUAC over time and is a useful way to identify unrealistic measurements in longitudinal measurements because changes to MUAC are limited by biological processes. While the literature on MUAC does not provide an absolute limit for MUAC velocity for children, the average reported values are in the range 0.14-0.43mm/day across several studies of inpatient (ITP) and outpatient therapeutic programs (OTP) for malnourished children (Chase et al. 2020; Goossens et al. 2012; Kamugisha et al. 2021; Tadesse et al. 2017). None of the children in this study were patients of ITC during data collection, but 61 of the 189 children were receiving therapeutic foods during the course of the project, which implies that they were patients of an OTP. In this sample of outpatient and non-patient children, we assume that the plausible range of MUAC velocity is ±0.7 mm/day. To put this figure in perspective, the maximum *average* velocity recorded in our review of the literature was 0.43 mm/day and in the only manuscript that used thresholds in MUAC velocity to identify implausible records, Schwinger, Fadnes, and Van den Broeck (2016) use 5cm/3months, which is approximately equal to 0.55 mm/day, assuming an average of 30.4 days per month; the threshold that we used is very generous.

While MUAC velocity identifies specific types of errors, it is not sensitive to all types of errors. We, therefore, use it simply to test if this type of errors is observed in the data submitted by participants. Our first approach for identifying low-quality observations was to calculate MUAC velocity and identify values greater than the ± 0.7 mm/day thresholds. The distributions of pooled velocities calculated from submissions are presented in Figure A1. The violation rate, the ratio of observations with velocities beyond the ± 0.7 mm/day thresholds, are also provided for the caregivers (left panel) and CHVs (right panel) in Figure A1. Analysis using thresholds at ± 0.6 mm/day and ± 0.8 mm/day were also used to test the results for robustness to changes in threshold. The results using the alternative thresholds were qualitatively identical to those presented here and are available upon request.

2.6% of the caregivers’ submissions violated the threshold and 16% of caregivers violate the threshold at least once. But 7% of them do so in more than 20% of their submissions. CHVs violate the velocity threshold less often, in only 0.8% of their observations. At the same time, 36% of CHVs violate the ±0.7 mm/day threshold at least once, but the maximum violation rate for a CHV is 4%. Put differently, there is evidence of errors in both the CHV and caregiver groups, but none of the CHVs have high rates (>20%) of error, while some of the caregivers do.

**Figure A1**. MUAC velocities from data submitted by caregivers (left panel) and from data submitted by CHVs (right panel), with the ±0.7mm/day thresholds indicated by the vertical lines.

| 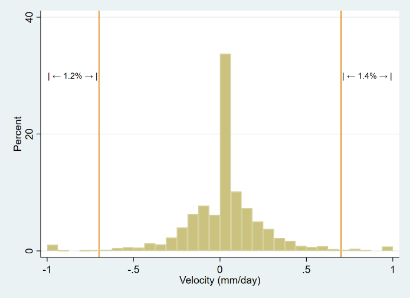 | 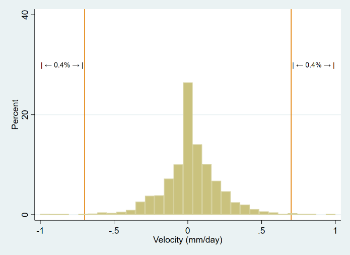 |
| --- | --- |
| Note: Velocities that were greater than 1 mm/day or less than -1 mm/day were recoded to 1 and -1, respectively. | |

Dropping those (unrealistic) observations that violate the 0.7mm/day threshold improves data quality, reducing the mean TEM significantly, from 0.68 to 0.56 (t-stat=2.31).

We next tested for differences in error rate, as indicated by MUAC velocity, between the participant and PIM generated data sets. For both the CHVs (Table A1, Model 1 vs. 2) and the caregivers (Table A1, Model 4 vs. 5), the PIM data had significantly fewer errors. Indeed, there are no velocity violations in the PIM-CHV dataset, although the rate of violations was quite low to begin with, less than 8 in 1,000. The PIM-caregiver data has a violation rate of 0.5%, less than 1/4^th^ the rate found in the full caregiver data. The differences between the caregiver and PIM-caregiver data are significantly different both in magnitude and statistical significance (difference=0.017, t-stat=3.16).

**Table A1**. Tests for differences in rates of MUAC velocity violations.

| Data source: | CHV data | PIM-CHV | CHV data with PIM | Difference (T-test) | | |
| --- | --- | --- | --- | --- | --- | --- |
| Model | (1) | (2) | (3) | (1)-(2) | (1)-(3) | (3)-(2) |
| Mean | 0.007 | 0 | 0.006 | 0.007*** | 0.001 | 0.006* |
| Standard Errors^1^ | [0.002] | [0] | [0.004] |  |  |  |
| t-statistic |  |  |  | 0.11 | 3.25 | 1.74 |
| Observations | 1,523 | 354 | 354 |  |  |  |
| Data source: | Caregiver data | PIM-Caregiver | Caregiver data with PIM | Difference (T-test) | | |
| Model | (4) | (5) | (6) | (4)-(5) | (4)-(6) | (6)-(5) |
| Mean | 0.022 | 0.005 | 0.006 | 0.018*** | 0.017*** | 0.001* |
| Standard Errors^2^ | [0.005] | [0.003] | [0.004] |  |  |  |
| t-statistic |  |  |  | 2.92 | 3.16 | 0.23 |
| Observations | 1,526 | 411 | 522 |  |  |  |
| Note: The value displayed for t-tests are the differences in the means across the groups. ^1^ Standard errors are clustered at the CHV level. ^2^ Standard errors are clustered at the caregiver level. ***, **, and * indicate significance at the 1, 5, and 10 percent critical level. | | | | | | |

As a final analysis of the data, we also test if the improvement in the data is due to the PIM process selecting for more accurate participant measurements. For example, it may be that the PIM process selects for measurements that were performed when children were sleeping (i.e., baby is still so it is easy to take a good photo, which is then more likely to be PIM-eligible). If so, it may be that the improvement in the data from the PIM process is mostly due to a selection effect rather than and actual difference between the participant measurements and PIM data. To test this, we compared the rate of velocity violations in the raw participant data to that of the sub-sample of raw participant data that was accompanied by an PIM-eligible photo. For the CHVs, there is little difference Table A1, (Model 1 vs. 3) but there is strong evidence of a selection effect for the caregivers (Table A1, Model 4 vs. 6).

We interpret these results to mean that PIM data can be used to increase the quality of the MUAC data sets and that these measurements are an improvement over measurements submitted directly from CHVs or caregivers. Furthermore, the improvement seems to originate from two sources. First, photos that meet the PI criteria require good light and a clear image of the measurement, which, for caregivers as least, seems to select for better measurement and/or recording conditions or greater effort by the participant. This selection process is able to screen out over 70% of the velocity violations in the raw caregiver data. Indeed, the added improvement of using the PIM data over the subset of caregiver data submitted with PIM-eligible photos is negligible. For CHVs, the situation is somewhat reversed but they start with already low rates of velocity errors. Our conclusion is that there is strong evidence that the PIM data contain fewer errors and some of that reduction comes from selection of better measurements by participant and some from improved interpretation or data recording of the measurement.

## References

Chase, Rachel P., Marko Kerac, Angeline Grant, Mark Manary, André Briend, Charles Opondo, and Jeanette Bailey. 2020. “Acute Malnutrition Recovery Energy Requirements Based on Mid-Upper Arm Circumference: Secondary Analysis of Feeding Program Data from 5 Countries, Combined Protocol for Acute Malnutrition Study (ComPAS) Stage 1.” *PloS One* 15(6):e0230452. doi: 10.1371/journal.pone.0230452.

Goossens, Sylvie, Yodit Bekele, Oliver Yun, Géza Harczi, Marie Ouannes, and Susan Shepherd. 2012. “Mid-Upper Arm Circumference Based Nutrition Programming: Evidence for a New Approach in Regions with High Burden of Acute Malnutrition.” *PLOS ONE* 7(11):e49320. doi: 10.1371/journal.pone.0049320.

Kamugisha, Jolly G. K., Betty Lanyero, Nicolette Nabukeera-Barungi, Harriet Nambuya-Lakor, Christian Ritz, Christian Mølgaard, Kim F. Michaelsen, André Briend, Ezekiel Mupere, Henrik Friis, and Benedikte Grenov. 2021. “Weight and Mid-Upper Arm Circumference Gain Velocities during Treatment of Young Children with Severe Acute Malnutrition, a Prospective Study in Uganda.” *BMC Nutrition* 7(1):26. doi: 10.1186/s40795-021-00428-0.

Schwinger, Catherine, Lars T. Fadnes, and Jan Van den Broeck. 2016. “Using Growth Velocity to Predict Child Mortality12.” *The American Journal of Clinical Nutrition* 103(3):801–7. doi: 10.3945/ajcn.115.118679.

Tadesse, Amare Worku, Elazar Tadesse, Yemane Berhane, and Eva-Charlotte Ekström. 2017. “Choosing Anthropometric Indicators to Monitor the Response to Treatment for Severe Acute Malnutrition in Rural Southern Ethiopia—Empirical Evidence.” *Nutrients* 9(12):1339. doi: 10.3390/nu9121339.

# Appendix B: Data collection

Following are a few additional details on the assumed costs:

- The annual and monthly data collections are performed by 7 enumerators, supported by one ILRI supervisor and one ILRI vehicle with a driver. Surveys are collected at the participants’ households. It takes the enumerator team 11 days to collect the 189 surveys.
- The daily rate for enumerators is set at the ILRI rate, which includes contributions to the national health care insurance fund and 15% additional fees charged by the firm to whom ILRI has subcontracted it’s hiring of short-term staff. The total cost to the project (not including ILRI’s own overheads) is $30/enumerator/day.
- For the cost of the Logiak software used by our Mbiotisho application, we have used the total cost for the 4-year contract between Logiak and FANRPAN, and divided it by 4, for the cost of the 12 months of data collection considered here. For the comparison groups, we have used the Dimagi Pro account for the 12 months of data collection.
- For the annual survey, the enumerators are trained before each round of the survey. For the monthly data collection, the enumerators are trained once at the beginning and there are four, one-day visits to the enumerators by the field supervisor to retrain and provide feedback. For the Mbiotisho application, there is a training for the contributors at the beginning of the activities, followed by four, one-day visits to the contributors by the field supervisor throughout the study, to retrain and provide feedback. But, in the case of Mbiotisho, each engagement is performed in groups of 48 participants, and must be repeated 4 times, which is how the actual implementation we performed.

Trainings for the Mbiotisho application are done locally so that there is no need to provide housing for contributors during training, while we do so for enumerators.

# Appendix C: Line-Item Budget for Data Collection Scenarios

|  | **Mbiotisho** | | |  | **Baseline/Endline** | | |  | **Monthly** | | |
| --- | --- | --- | --- | --- | --- | --- | --- | --- | --- | --- | --- |
|  | Units | $/unit | Total |  | # Units | $/unit | Total |  | # Units | $/unit | Total |
| **Training 1** |  |  |  |  |  |  |  |  |  |  |  |
| Food lodging & training location for trainees for 6-day training | 1,134 | 15 | 17,010 |  | 42 | 35 | 1,470 |  | 42 | 35 | 1,470 |
| Stipend for trainees for 6 days | 1,134 | 5 | 5,670 |  | 42 | 30 | 1,260 |  | 42 | 30 | 1,260 |
| Vehicle | 1,300 | 1.07 | 1,391 |  | 900 | 1.07 | 963 |  | 900 | 1.07 | 963 |
| Driver per diem & lodging (+2 days travel/prep) | 32 | 65 | 2,080 |  | 16 | 65 | 1,040 |  | 16 | 65 | 1,040 |
| Field supervisor per diem & lodging (+2 days travel/prep) | 32 | 65 | 2,080 |  | 16 | 65 | 1,040 |  | 16 | 65 | 1,040 |
|  |  |  |  |  |  |  |  |  |  |  |  |
| **Maintenance & retraining, 4 1-day engagements** |  |  |  |  |  |  |  |  |  |  |  |
| Food lodging & training location for trainees | 756 | 15 | 11,340 |  |  |  |  |  | 28 | 35 | 980 |
| Stipend for trainees | 756 | 5 | 3,780 |  |  |  |  |  | 28 | 30 | 740 |
| Vehicle | 1,300 | 1.07 | 1,391 |  |  |  |  |  | 900 | 1.07 | 963 |
| Driver per diem & lodging (+2 days travel/prep) | 32 | 65 | 2,080 |  |  |  |  |  | 12 | 65 | 780 |
| Field supervisor per diem & lodging (+2 days travel/prep) | 32 | 65 | 2,080 |  |  |  |  |  | 12 | 65 | 780 |
|  |  |  |  |  |  |  |  |  |  |  |  |
| **Endline Training** |  |  |  |  |  |  |  |  |  |  |  |
| Food lodging & training location for trainees |  |  |  |  | 42 | 35 | 1,470 |  |  |  |  |
| Stipend for trainees |  |  |  |  | 42 | 30 | 1,260 |  |  |  |  |
| Vehicle |  |  |  |  | 900 | 1.07 | 963 |  |  |  |  |
| Driver per diem & lodging (+2 days travel/prep) |  |  |  |  | 8 | 65 | 520 |  |  |  |  |
| Field supervisor per diem & lodging (+2 days travel/prep) |  |  |  |  | 8 | 65 | 520 |  |  |  |  |
|  |  |  |  |  |  |  |  |  |  |  |  |
| **Hardware** |  |  |  |  |  |  |  |  |  |  |  |
| Phones | 189 | 100 | 18,900 |  |  |  |  |  |  |  |  |
| Solar Chargers | 189 | 25 | 4,725 |  | 7 | 25 | 175 |  | 7 | 25 | 175 |
| Tablets |  |  |  |  | 7 | 350 | 2,450 |  | 7 | 350 | 2,450 |
|  |  |  |  |  |  |  |  |  |  |  |  |
| **Software costs** |  |  |  |  |  |  |  |  |  |  |  |
| Logiak | 0.25 | 81,000 | 20,250 |  |  |  |  |  |  |  |  |
| Dimagi |  |  |  |  | 12 | 500 | 6,000 |  | 12 | 500 | 6,000 |
|  |  |  |  |  |  |  |  |  |  |  |  |
| **Data collection** |  |  |  |  |  |  |  |  |  |  |  |
| Participant token | 59,947 | 0.20 | 11,989 |  | 378 | 5 | 1,890 |  | 2,268 | 5 | 11,340 |
| Airtime & Data | 2,268 | 5 | 11,340 |  | 18 | 25 | 450 |  | 108 | 25 | 2,700 |
| Enumerators lodging and food |  |  |  |  | 154 | 35 | 5,390 |  | 924 | 35 | 32,340 |
| Enumerator pay |  |  |  |  | 154 | 30 | 4,620 |  | 924 | 30 | 27,720 |
| Vehicle |  |  |  |  | 1,800 | 1.07 | 1,926 |  | 10,800 | 1.07 | 11,556 |
| Driver per diem & lodging (+2 days travel/prep) |  |  |  |  | 26 | 65 | 1,690 |  | 156 | 65 | 10,140 |
| Field supervisor per diem and lodging (+2 days travel/prep) |  |  |  |  | 26 | 65 | 1,690 |  | 156 | 65 | 10,140 |
|  |  |  |  |  |  |  |  |  |  |  |  |
| **Staff time** |  |  |  |  |  |  |  |  |  |  |  |
| Research officer, active during training, maintenance, and data collection | 32 | 280 | 8,960 |  | 42 | 280 | 11,760 |  | 170 | 280 | 47,600 |
|  |  |  |  |  |  |  |  |  |  |  |  |
| **Total** |  |  | **125,066** |  |  |  | **48,547** |  |  |  | **172,277** |
